# *Pestivirus K* (Atypical Porcine Pestivirus): Update on the Virus, Viral Infection, and the Association with Congenital Tremor in Newborn Piglets

**DOI:** 10.3390/v12080903

**Published:** 2020-08-18

**Authors:** Alais M. Dall Agnol, Alice F. Alfieri, Amauri A. Alfieri

**Affiliations:** 1 Laboratory of Animal Virology, Department of Veterinary Preventive Medicine, Universidade Estadual de Londrina, Londrina, CEP 86057-970 Paraná, Brazil; alaisagnol@hotmail.com (A.M.D.A.); aalfieri@uel.br (A.F.A.); 2 Multi-User Animal Health Laboratory, Molecular Biology Unit, Department of Veterinary Preventive Medicine, Universidade Estadual de Londrina, CEP 86057-970 Paraná, Brazil; 3 Rodovia Celso Garcia Cid Road-Campus Universitário, Londrina, PO Box 10011, CEP 86057-970 Paraná, Brazil

**Keywords:** APPV, pestiviruses, congenital tremor type A-II, persistent infection, pigs

## Abstract

The atypical porcine pestivirus (APPV) belongs to the species *Pestivirus K* of the genus *Pestivirus* and the family *Flaviviridae*, and it has been associated with congenital tremor (CT) type A-II in newborn piglets. Although APPV was discovered in 2015, evidence shows that APPV has circulated in pig herds for many years, at least since 1986. Due to the frequently reported outbreaks of CT on different continents, the importance of this virus for global pig production is notable. Since 2015, several studies have been conducted to clarify the association between APPV and CT. However, some findings regarding APPV infection and the measures taken to control and prevent the spread of this virus need to be contextualized to understand the infection better. This review attempts to highlight advances in the understanding of APPV associated with type A-II CT, such as etiology, epidemiology, diagnosis, and control and prevention measures, and also describes the pathophysiology of the infection and its consequences for pig production. Further research still needs to be conducted to elucidate the host’s immune response to APPV infection, the control and prevention of this infection, and the possible development of vaccines.

## 1. Introduction

In 2015, the atypical porcine pestivirus (APPV) was first described and identified by next-generation sequencing (NGS) in pig serum samples [1]. Initially, the virus was not believed to be associated with any clinical manifestations; later, an experimental inoculation study conducted in the United States (US) demonstrated that APPV was associated with the occurrence of congenital tremor (CT) in newborn piglets [2]. At present, the occurrence of APPV associated with CT is frequently reported by several studies on different continents, including the Americas (North and South), Europe, and Asia [3,4].

Since 2015, several studies have been conducted to clarify the association between APPV and CT. However, several findings regarding the infection and the measures taken to control and prevent the spread of the virus need to be contextualized to characterize the infection better. This review aims to address advances in understanding APPV associated with CT type A-II and further describe the pathophysiology of this virus and its consequences for pig production.

## 2. Congenital Tremor Syndrome

CT is a neurological disorder that affects newborn piglets and is characterized by muscle spasms in the head and body, which can be localized or generalized [5,6]. The intensity of the tremors is variable, and in more severe cases, they include generalized tremors that result in difficulty in standing or walking and, consequently, inability to suckle, resulting in death due to starvation. The clinical manifestation of CT is also called shaker pig syndrome, trembling pigs, or congenital myoclonus [6,7].

CT syndrome is classified into A and B according to the presence or absence of histopathological lesions in the central nervous system (CNS). In CT type A, histopathological changes are found in the brain or spinal cord. When histopathological lesions are not observed, it is classified as CT type B. Based on the etiology of CT type A, this type is subdivided into five subgroups (I-V) [5]. CT type A-I is characterized by cerebellar hypoplasia, dysplasia, hypomyelination of the spinal cord, and its etiology associated with other porcine pestivirus infections in the classical swine fever virus (CSFV) [8].

For a long time, the cause of CT A-II was not defined, although its infectious etiology has always been related to this type of tremor [9]. Since 2016, the etiology of CT A-II has been attributed to the newly described APPV, which has already been demonstrated by experimental infections [2,10]. Other viruses also were detected coinfection with APPV, such as porcine circovirus type 2 [11], astrovirus [12], porcine circovirus type 3 [13], linda virus [14], porcine circovirus-like virus P1 [15], and *Teschovirus A* [16], however, the role played by these three viruses as the primary cause of CT A-II is debatable.

CT type A-III is related to a genetic defect presented only by the Landrace breed, in which a lack of myelin is observed together with a reduction in the number of oligodendrocytes [17]. CT type A-IV is caused by a recessive genetic defect in the Saddleback swine and is characterized by hypomyelination of the brain and spinal cord [18,19]. Type A-V is caused by metrifonate (trichlorfon) intoxication. The occurrence of this type of CT may be associated with the use of metrifonate as an antiparasitic medication, and when administered during pregnancy, CT type A-V can cause cerebellar hypoplasia and cause piglets to be born with ataxia and tremor [20,21]. Finally, CT type B has no known specific cause, and changes in the CNS are not observed [5].

## 3. Atypical Porcine Pestivirus

### 3.1. Classification and Etiology

Until 2017, only four “classical” species belonged to the genus *Pestivirus*: bovine viral diarrhea virus 1 (BVDV-1); bovine viral diarrhea virus 2 (BVDV-2); border disease virus (BDV); and CSFV [22]. However, a large number of viruses linked to pestiviruses have been described [1,23,24,25,26,27,28,29,30,31,32], and Smith et al. [22] proposed that the study group of the *Flaviviridae* family of the International Committee on Viral Taxonomy (ICTV) revise the taxonomy of the genus *Pestivirus*. Thus, the genus *Pestivirus* was divided into 11 species, *Pestivirus A* (BVDV-1) [33], *Pestivirus B* (BVDV-2) [34], *Pestivirus C* (CSFV) [35], *Pestivirus D* (BDV) [36], *Pestivirus E* (pronghorn antelope pestivirus) [29], *Pestivirus F* (porcine pestivirus) [26], *Pestivirus G* (giraffe pestivirus) [23], *Pestivirus H* (HoBi-like pestivirus) [27], *Pestivirus I* (Aydin-like pestivirus) [24], *Pestivirus J* (rat pestivirus) [25], and *Pestivirus K* (atypical porcine pestivirus) [1], and one unclassified virus, bat pestivirus [30].

APPV is the single species of *Pestivirus K*, to the genus *Pestivirus*, and to the family *Flaviviridae* [37]. The viral particle is spherical, with a diameter of approximately 60 nm [38] and enveloped. Viral genomes are single-stranded, positive-sense RNA, exhibiting a genome size of approximately 11 to 11.6 kb. The genome comprises a 5′-noncoding region (NCR), one single open reading frame region encoding a single polyprotein with 3635 amino acids, and a 3′-NCR region [1,39]. The polyprotein is processed into 12 proteins, C (capsid protein), E^rns^, E1, E2 (envelope proteins), and nonstructural proteins N^pro^, p7, NS2, NS3, NS4A, NS4B, NS5A, and NS5B [40].

Phylogenic analyzes of the complete or partial genome of APPV strains detected in different countries and years, and in pigs from commercial farms and wild boars, demonstrate a high degree of genetic variability, subdividing into different clusters [3,41,42,43,44].

### 3.2. Pathogenesis and Clinical-Pathological Manifestation Associated with APPV

Two studies of experimental infection were carried out to associate APPV infection with CT and elucidate the pathogenesis. The first experimental inoculation was performed by Arruda et al. [2] and occurred in the US. Five pregnant sows, a group at 45 days and another at 62 days of gestation, were inoculated in the fetal amniotic vesicle, intranasally, and intravenously with known serum samples positive for APPV. In the period following the inoculation, no inoculated sow showed detectable viremia by qRT-PCR and no observation of clinical signs suggestive of CT. After birth, piglets were monitored for CT, and 57% to 100% of piglets showed signs of tremor. In the second experimental study carried out in the Netherlands by de Groof et al. [10], APPV-positive sera were inoculated intramuscularly in three gilts at 32 days of gestation. At 10 days postinfection, the sera from three gilts were RT-PCR-positive for the virus, and one of the gilts presented a relatively lower viral load than the other gilts. At birth, two of the three litters contained piglets showing clinical signs of CT, ranging from mild to severe signs in almost all piglets. No piglets born from the gilt that had low viremia showed signs of CT.

In both experimental studies, clinical signs were classified according to intensity; the most severe cases were characterized by intense tremors throughout the body, while the mild ones were characterized by muscle fasciculation in the limbs. Another clinical sign frequently observed was the presence of piglets with the splay leg [2,10]. This clinical manifestation is a syndrome characterized by temporary dysfunctionality of the posterior leg muscles, occurring shortly after birth, and resulting in difficulty or inability to stand and walk [45]. The presence of APPV RNA was detected in different organs of all piglets that showed signs of CT, and in some of the piglets not affected by CT [2,10]. These experiments were essential to elucidate the association of APPV with CT. However, Koch’s postulate was not fulfilled by the two studies, probably due to the difficulty of viral isolation in cell cultures.

In the various studies that describe natural and experimental infections, the virus can be detected by conventional RT-PCR, qRT-PCR, and immunohistochemical in a wide variety of organs. The highest viral loads are observed primarily in the CNS and lymphoid tissues. In the CNS, the virus is found in greater quantities in the cerebellum and lymphoid tissues, mainly in the inguinal lymph nodes, submandibular lymph nodes, and thymus, suggesting that the cerebellum and lymph nodes are target organs of APPV [46,47]. In other organs, such as the brain, brain stem, spinal cord, heart, liver, spleen, lungs, and kidneys, the viral load is relatively lower [42,46,47].

To date, no significant macroscopic lesions have been described in piglets with CT [48,49,50]. Histopathological findings in APPV infections primarily include vacuolization of the white matter in the cerebellum [48,50,51]. Studies using Luxol^®^ Fast Blue staining have shown a reduction in the intensity of myelin staining in the white matter of the spinal cord, cerebrum, cerebellum, and sciatic nerve, which implies a decrease in the amount of myelin in these tissues [16,48,50,52,53]. Gliosis has also been observed in the cerebral cortex and, to a lesser extent, in the spinal cord [16,49,50]. Other histopathological lesions observed in affected piglets were neuronal necrosis, neuronophagia with satellitosis, particularly at the cerebral cortex and spinal cord, Wallerian degeneration of the spinal cord, and necrosis of Purkinje cells of the cerebellum [49]. Transmission electron microscopy performed in two CNS tissues revealed not only hypomyelination of the cerebellar white matter but also more severe changes, such as interruption and breakdown of myelin, in animals affected by APPV [50]. These histopathological lesions described in piglets with CT, primary demyelination, and/or hypomyelination, can be considered a neuropathological characteristic associated with APPV infection [49]. The high viral loads of APPV in the CNS, along with the lesions observed in these tissues, explains the intensity of the neurological symptoms caused by APPV [46,47].

### 3.3. Viral Shedding and Transmission

Piglets that developed CT and recovered were monitored by some studies to assess the possibility of viral excretion and duration. Therefore, de Groof et al. [10] followed five pigs that presented CT and recovered at 8.5 months of age; these animals continued to shed APPV in their feces. Excretion in feces was also suggested by Postel et al. [53] since high viral loads were detected in the duodenum, pancreas, and colon. APPV is found in salivary glands; therefore, the virus is shedding by saliva until at least the sixth month of life [50]. In these same animals, the virus was also found in semen. Another study evaluated semen collection from three commercial boar herds and detected the presence of the viral genome in swab and fluid preputial and in the semen from 15% of boars from a commercial boar stud, suggesting that artificial insemination may be an important form of transmission [54].

Regarding transmission, the horizontal and vertical forms of transmission were described, with the latter being the most important and responsible for the development of CT in newborn piglets from naturally infected sows. Transplacental transmission was demonstrated by experimental inoculation. Pregnant sows were infected with serum samples known to be positive for APPV, and the birth of piglets with CT of different levels of severity was observed [2,10].

In a controlled exposure, using fetal fluid from litters that had exhibited CT, a batch of 91 gilts was submitted to oral exposure to the antigen approximately 54 days before breeding. Signs of CT were observed in 45.0% of the litters and 30.8% of all piglets born [51]. These data suggest probable horizontal transmission, due to oral exposure, and the development of CT cases.

In piglets, horizontal transmission was described after the monitoring of piglets that showed signs of CT and obtained a confirmed diagnosis for APPV. After weaning, naive piglets were mixed with APPV-positive piglets. Due to close contact, infection of susceptible piglets occurred, suggesting that transmission occurs after mixing infected with susceptible piglets. No manifestation of disease was observed in this type of late infection; moreover, these animals were transiently infected [55]. It is likely that this type of transmission is related to environmental contamination and implies the spread of the virus. However, little is known about the forms of transmission and their possible effects; therefore, further investigations are necessary for a satisfactory clarification on this topic.

### 3.4. Epidemiology and Implications in Pig Production

In the United States, APPV was first identified by NGS in pig serum samples collected in 2014 from five different states [1]. In subsequent years, several studies from different countries reported the circulation of APPV in the US [1,2,51,54,56], Germany [53,55,57,58,59,60,61], Netherlands [10], Sweden [62], Austria [50], England [63], China [38,42,44,46,47,61,64,65,66,67,68,69,70,71,72,73], Spain [41,74], South Korea [75], Brazil [16,48,49,76], Great Britain [61], Italy [43,61], Serbia [61], Switzerland [61,77], Taiwan [61], Canada [52], and Hungary [78].

Epidemiological surveys demonstrate a high number of the pigs positive for APPV, depending on the region evaluated and the assay used. Postel and colleagues [61] used qRT-PCR to analyze 1460 serum samples from asymptomatic pigs from Germany, Italy, Serbia, Great Britain, Switzerland, China, and Taiwan and found a prevalence of 8.9% (130/1460) of APPV-positive samples. Additionally, in this same study, the highest (17.5%; 35/200) prevalence of positive samples was in Italy. Michelitsch et al. [59], by indirect immunofluorescence test (IFI), found an antibody prevalence of 16.3% (182/1115) in pig serum samples in Germany. In Switzerland, 1080 sera from pigs for slaughter, which were obtained between 1986 and 2018, were evaluated by qRT-PCR, and the APPV genome was detected in 13% [77].

Although this virus was discovered recently, some studies have shown that APPV has been circulating in pig herds for a long time. A study conducted in Spain detected the presence of the viral genome in pig serum samples collected in 1997 [41]. In Switzerland, another investigation analyzed 1080 pig sera collected between 1986 and 2018 and revealed that 7% (6/87) of the samples collected in slaughter pigs in 1986 were APPV-positive [77]. These findings correspond to reports of the long-term presence of APPV in the world.

The presence of APPV in wild boars has been demonstrated in Germany, Spain, and Italy, and the prevalence of the virus varies according to the country. In Germany, 456 wild boar serum samples were analyzed, and 19% of samples revealed the presence of the viral genome, while 52% of the serum samples had antibodies against the E^rns^ protein [50]. In Spain and Italy, the prevalence observed was lower, 0.23% (1/437), and 0.69% (3/430) of the evaluated sera had the virus genome, respectively [43,74]. Transmission of APPV from wild boars to pigs or vice versa has not been demonstrated to date. However, wild boars are sources of various pathogens that infect domestic pigs, including other pestiviruses, such as CSFV [79], and can contribute to their transmission. Due to the importance of the transmission of viral pathogens, these wild animals are a challenge for pig health and must be considered for effective control plans to be devised.

Regarding the occurrence of CT, the litters from first parity sows are most affected by APPV infection, and this infection rarely occurs in higher birth order [10,48,49]. During an outbreak on a farm in the Netherlands, 48 litters with CT were born from gilts and were monitored, the number of piglets affected within each litter ranged from <10 to 100%. Furthermore, the total mortality of piglets, reached 26%, with 60% of these deaths being attributed to CT [10]. In Brazil, an outbreak of CT observed in piglets born to gilts, lasted for three weeks, and the mortality rate reached 30% [49].

### 3.5. APPV Infection and Immunity

The dynamics of APPV infection can be hypothesized in two ways: persistently infected and transiently infected animals. These forms of infection are known to other pestiviruses, as is the case with CSFV [79] and BVDV in cattle [80]. In APPV infection, the dynamics have not been fully elucidated; however, this phenomenon can be explained by two studies [50,55]. Schwarz et al. [50] monitored the health status of two piglets (one female and one male) aged up to six months; shortly after birth, these two animals showed CT. Specific antibodies to APPV NS3 were detected at birth and at up to eight weeks of age. The tremor symptoms decreased and disappeared completely until 14 weeks; however, both piglets still presented viremia, antibody titer, and shedding of the virus by saliva. At six months the male piglet reached sexual maturity, and a high viral load was detected in saliva and semen; on the other hand, viremia was reduced.

Cagatay et al. [55], using direct and indirect tests, monitored 20 piglets from unaffected and affected litters by CT from birth to slaughter. In the vertically infected and symptomatic piglets, viremia was detected from the first days of life until slaughter. For the presence of antibodies, these piglets showed high levels of antibodies at six days of age, and these antibodies were undetectable at 21 and/or 48 days of age. It is possible that these antibodies came from the sows and disappeared with the drop in passive immunity. On the other hand, piglets infected horizontally after being mixed at weaning with those infected vertically showed viremia at 48 days of age and high titers of specific antibodies to E2 when evaluated at 69 and 161 days of life, suggesting the induction of protective immunity against infection.

Based on piglets infected horizontally, the immune response was higher for the E2 protein, and neutralizing antibody titers correlated with the presence of E2-specific antibodies, while a correlation with E^rns^-specific antibodies was not observed [58].

Due to the longevity of viral shedding, detectable viremia, together with the disappearance of specific antibodies over time, may suggest that persistent infection (PI) can be attributed to piglets that are intrauterine-infected. Soon after birth, these piglets can show signs of CT, which usually regresses over time [41,50,61]. On the other hand, piglets infected horizontally, through contact with persistently infected animals, develop a transient infection, with viremia detected for several weeks, but over time the piglet develops active immunity against APPV, and the virus becomes undetectable [55]. These studies provide evidence of the dynamics of infections and the immune response; however, the studies examine a small number of animals, meaning that further studies are required for complete elucidation.

### 3.6. Diagnostic Methods of APPV

Currently, a wide variety of diagnostic techniques are available for use in elucidating APPV infection in pigs. Due to practicality, speed, sensitivity, and specificity, molecular tests are the most commonly used. Among these tests, both conventional RT-PCR [16,48,49] and qRT-PCR [2] have been described in viral detection in several studies. Different clinical samples can be subjected to viral detection. In clinically affected animals, the CNS and the lymphoid organs are specimens of choice, since higher viral loads are found in these tissues, especially the cerebellum and lymph nodes [46,47]. Another available technique is NGS [2,10,51], which has been used since the first viral description in 2015 [1], and NGS is still employed as a diagnostic tool, assisting in the detection of the viral genome. In addition, NGS provides data on possible coinfections and primarily obtains larger fragments of the viral genome, which favors the phylogenetic study of circulating strains.

Histological tests performed from tissues fixed in paraffin are of great diagnostic importance, enabling the visualization of lesions caused by APPV. Luxol^®®^ Fast Blue staining helps to observe the demyelination caused by the APPV in the CNS, primarily located in the cerebellum and spinal cord [16,48,50,52,53]. Histopathology, together with an immunohistochemical technique [46], and in situ hybridization [53,60] enables the detection of the viral agent (protein or nucleic acid) at the lesion site.

Other important methods that are used to assist in the diagnosis of infectious diseases are serological tests. The tests described for APPV infection are IFI [59], virus neutralization [55], and indirect enzyme-linked immunosorbent assay (ELISA) to the NS3, E2, and E^rns^ proteins [50,55,58,61]. These tests have many important applications, and they can be used for population-based epidemiological studies and monitoring of infection in the herd; the tests also feature easy execution and low cost.

### 3.7. Control and Prevention

To date, there are no effective drugs or vaccines available to treat or prevent APPV infection. Zhang et al. [81] constructed a recombinant baculovirus of APPV glycoprotein E2, which induced a robust humoral and cellular immune response in mice. Based on these studies and knowing that the E2 protein is responsible for inducing neutralizing antibodies [55], this vaccine appears to be a promising tool as likely prevention of APPV in pigs. However, further viral challenge studies are needed to demonstrate an effective immune response.

As litters of gilts are most affected by APPV infection [10,49], it is probable that the introduction of naive gilts in the herd is an important issue in the epidemiology of the disease [49,55]. Therefore, preventive measures are necessary to address this issue. The use of acclimatization for replacement gilts is an interesting tool that can be used in diseases that do not have vaccines and/or when available are not completely effective for prevention. Gatto et al. [3] suggest the protocol similar to what is used for the control of enzootic pneumonia caused by *Mycoplasma hyopneumoniae*. However, once horizontal transmission in gilts after oral exposure to fetal fluid antigen positive for APPV has been demonstrated, followed by the induction of CT in the litters of these gilts [51], this measure needs to be considered carefully. There is a need to conduct studies focused on this tool and, therefore, the development of protocols aimed at infection by APPV.

Another important point to be considered when designing control programs is the possibility of PI animals. Sensitive diagnostic methods that are able to detect PI animals, such as qRT-PCR, especially in the case of breeders, are highly important. After identifying these animals, they must be removed from the herd due to viral shedding by semen, feces, and oral fluid, which favors viral transmission. Another issue to be monitored is the semen used in the practice of artificial insemination; one possible measure is to include testing for APPV in the routine tests performed on these boars and their semen.

Despite specific measures to control APPV, biosecurity measures cannot be overlooked. When replacing breeders, animals from herds with a good health strategy must be purchased, and quarantining must be performed before introduction on the farm. Traffic control of people and vehicles on farms must be considered in addition to the execution of adequate cleaning and disinfection programs in the facilities. Finally, the control of wild animals, rodents, and insects contributes to the health of the herd in general.

## 4. Conclusions and Perspectives

The new porcine pestivirus (APPV) is strongly related to cases of CT type A-II, which until 2016, had an undefined etiology. Despite the recent discovery of APPV, evidence has shown that this virus has been circulating in pig herds for many years, since at least 1986, along with longstanding reports of CT, and the importance of this virus for global pig production is notable. In addition, APPV belongs to the genus *Pestivirus*, presenting important biological characteristics for the epidemiology of the disease, such as viral persistence, which can represent viral maintenance and a constant source of both horizontally and vertically transmitted infection in pig herds. These points are highly important for viral prevention. However, these issues and others regarding this disease still need to be elucidated, and further research should investigate the host’s immune response, the control and prevention of APPV infection, and the development of vaccines.
